# A Morphological Study of Solvothermally Grown SnO_2_ Nanostructures for Application in Perovskite Solar Cells

**DOI:** 10.3390/nano12101686

**Published:** 2022-05-15

**Authors:** Zhuldyz Yelzhanova, Gaukhar Nigmetova, Damir Aidarkhanov, Bayan Daniyar, Bakhytzhan Baptayev, Mannix P. Balanay, Askhat N. Jumabekov, Annie Ng

**Affiliations:** 1 Department of Electrical and Computer Engineering, School of Engineering and Digital Sciences, Nazarbayev University, Kabanbay Batyr Ave. 53, Nur-Sultan 010000, Kazakhstan; zhuldyz.yelzhanova@nu.edu.kz (Z.Y.); aidarkhanov@nu.edu.kz (D.A.); bayan.daniyar@nu.edu.kz (B.D.); 2 Department of Chemical and Materials Engineering, School of Engineering and Digital Sciences, Nazarbayev University, Kabanbay Batyr Ave. 53, Nur-Sultan 010000, Kazakhstan; gaukhar.nigmetova@nu.edu.kz; 3 National Laboratory Astana, Kabanbay Batyr Ave. 53, Nur-Sultan 010000, Kazakhstan; bbaptayev@nu.edu.kz; 4 Department of Chemistry, School of Sciences and Humanities, Nazarbayev University, Kabanbay Batyr Ave. 53, Nur-Sultan 010000, Kazakhstan; mannix.balanay@nu.edu.kz; 5 Department of Physics, School of Sciences and Humanities, Nazarbayev University, Kabanbay Batyr Ave. 53, Nur-Sultan 010000, Kazakhstan; askhat.jumabekov@nu.edu.kz

**Keywords:** Tin(IV) oxide, nanorods, nanostructures, solvothermal growth, growth parameters, electron transport layer, perovskite solar cells

## Abstract

Tin(IV) oxide (SnO_2_) nanostructures, which possess larger surface areas for transporting electron carriers, have been used as an electron transport layer (ETL) in perovskite solar cells (PSCs). However, the reported power conversion efficiencies (PCEs) of this type of PSCs show a large variation. One of the possible reasons for this phenomenon is the low reproducibility of SnO_2_ nanostructures if they are prepared by different research groups using various growth methods. This work focuses on the morphological study of SnO_2_ nanostructures grown by a solvothermal method. The growth parameters including growth pressure, substrate orientation, DI water-to-ethanol ratios, types of seed layer, amount of acetic acid, and growth time have been systematically varied. The SnO_2_ nanomorphology exhibits a different degree of sensitivity and trends towards each growth factor. A surface treatment is also required for solvothermally grown SnO_2_ nanomaterials for improving photovoltaic performance of PSCs. The obtained results in this work provide the research community with an insight into the general trend of morphological changes in SnO_2_ nanostructures influenced by different solvothermal growth parameters. This information can guide the researchers to prepare more reproducible solvothermally grown SnO_2_ nanomaterials for future application in devices.

## 1. Introduction

Today we are facing a high demand for energy sources due to the continuous increase of the human population and ever-advancing technologies. The huge energy consumption cannot rely only on the non-sustainable supplies from conventional energy. The development of renewable energy sources is of significant importance to relieve the energy burden and minimize the release of pollutants during energy generation. Recently, perovskite solar cells (PSCs) have made a breakthrough in the field of photovoltaics (PVs). In the last decade, the power conversion efficiency (PCE) of PSCs has increased from the first reported value of 3.8% to the latest record of 25.7% [1]. Such rapid growth of the PCE of PSCs is due to the impressive intrinsic properties of halide perovskite absorber materials with a high absorption coefficient, high charge carrier mobility, tunable bandgap, long charge carrier diffusion lengths, etc. [2]. Nevertheless, a number of challenges, such as material stability, the device lifetime, scalability, and the toxicity of Pb-based perovskites, should be well addressed before PSCs enter the stage of commercialization [3,4,5,6,7]. Over the past decade, a variety of research areas, including compositions of halide perovskite [8], perovskite growth techniques [9,10], architectures of PSCs, and bulk and interface passivation engineering [11,12,13], as well as the optimization of different functional layers of PSCs [14,15,16], have made significant breakthroughs. The promising results continuously achieved in the community provide the fundamentals for accelerating the advancement of photovoltaic technologies for future practical PSCs.

The performance of charge-transporting layers is one of the crucial factors in determining the efficiency and stability of PSCs. Nowadays, the majority of electron transport layers (ETLs) used in PSCs are based on the transition metal oxides such as TiO_2_, SnO_2_, and ZnO. The active research endeavors have been devoted to the optimization of the morphology of ETLs and its interface for subsequent perovskite deposition [17,18,19,20], enhancement of the ETL electronic properties for efficient electron transports [21], and development of transition metal oxide nanostructures for facilitating carrier extraction [22]. The impacts of the ETLs in nanostructures on carrier transport properties and device performance are shown to be different compared to the ETLs in the form of thin films. It is believed that an ETL in nanostructures has a larger surface area, which can facilitate the interfacial charge transfer between the photo-absorber and the photoanode. Therefore, it becomes interesting to investigate the performance of PSCs incorporating ETLs composed of different nanostructures [23]. Inherited from the architecture of dye-sensitized solar cells (DSSCs), the ETLs based on TiO_2_ are the earliest type of transition metal oxide used in PSCs. Nowadays, TiO_2_ in compact and mesoscopic structures is commonly employed in PSCs, generating high PCE records. Owing to the high versatility of preparation techniques, a variety of TiO_2_ nanostructures, such as nanorods [24,25,26,27,28,29,30,31,32,33,34], nanowires [35,36,37,38], nanotubes [39], nanosheets [40], and other complex nanostructures, such as nanoflowers [41] and hierarchical TiO_2_ nanostructures, have been synthesized and demonstrated as ETLs employed in PSCs. However, the strong photocatalytic activities of TiO_2_ are concerning as it is believed to be one of the factors leading to the quick degradation of PSCs under long-term illumination. The ZnO material is an alternative for the TiO_2_ ETL used in PSCs. It has higher charge mobility compared to TiO_2_ and the processing temperatures for preparing ZnO thin films and nanostructures are relatively lower than that of TiO_2_, which is significantly important to the development of flexible PSCs for portable and wearable electronics. The ZnO nanoparticles [42] and nanorods [43,44,45,46,47,48,49,50,51] are the common nanostructures used for electron-transporting materials in PSCs. However, so far, the reported PCEs of ZnO-based PSCs are lower than that of PSCs with TiO_2_ as the ETLs. This is possibly due to large surface defect concentrations of ZnO [52], and deprotonation of methylammonium of the perovskite materials in contact with ZnO, which limits the thermal annealing temperatures and duration of annealing time, likely leading to incomplete conversion of the precursor materials to perovskites and formation of perovskite thin films with low crystallinity. Therefore, the use of ZnO as the ETL in PSCs requires additional considerations for selecting compatible perovskite materials (e.g., all-inorganic-based perovskites) as well as device-processing conditions. Recently, SnO_2_ materials have become a popular option for ETL used in PSCs due to a number of advantages. It has a higher electron mobility (around 100–250 cm^2^/V·s) compared to TiO_2_ (<1 cm^2^/V·s) [53] and possesses a deep conduction band, aligning well with the perovskites, leading to a higher open-circuit voltage (*V_OC_*) of PSCs. Meanwhile, SnO_2_ is not an active photocatalytic material, which is one of the desired properties for the chemical stability of the photovoltaic materials as well as the lifetime of the devices. Same as ZnO, the SnO_2_-based ETL can be also prepared by different low-temperature processing techniques, which are advantageous to the development of flexible PSCs. Recently, the SnO_2_ material used as an ETL in PSCs, yielding high PCEs, is usually in the form of compact thin film and nanoparticles [12]. The PSCs based on other nanostructured SnO_2_ ETLs have also been demonstrated [54,55,56,57,58,59,60,61,62,63], while their reported photovoltaic performance has a huge deviation in different works [55,56,57]. This is probably due to the large degree of variation in nanomorphology of ETLs controlled by multiple material growth parameters, causing the complexity in finding the optimal processing conditions for the fabrication of PSCs. The SnO_2_ nanostructures can be synthesized in an autoclave reactor via the solvothermal growth method [64]. This technique has gained great success in preparing nanomaterials due to its advantages, such as low processing temperature and energy consumption, low-cost manufacturing (i.e., equipment, raw materials etc.), and the environmental benignity of the process. In this work, the solvothermal technique for preparing SnO_2_ nanorod arrays is systematically investigated. The impact of different growth parameters including the (i) growth pressure, (ii) substrate orientation, (iii) deionized (DI) water-to-ethanol ratios, (iv) types of seed layer, (v) amount of acetic acid, and (vi) growth time on the morphology of SnO_2_ nanostructures are studied. The SnO_2_ nanorod arrays prepared under different conditions are characterized. The obtained results provide thorough information to the community for synthesizing SnO_2_ nanostructures with improved reproducibility for the application in PSCs.

## 2. Experimental Details

### 2.1. Synthesis of SnO_2_ Nanorod Arrays

Fluorine-doped tin oxide (FTO)-coated glasses were used to grow SnO_2_ nanorod arrays. The substrates were cleaned by a thorough cleaning procedure using acetone, isopropanol (IPA), and DI water in an ultrasonic bath. The cleaned substrates were air-dried by nitrogen and then put in an ultraviolet ozone cleaning system (UVOCS) for 30 min to eliminate organic contaminants. Unless otherwise specified, the precursor solution of SnO_2_ was prepared by dissolving 0.15 mmol sodium bromide (NaBr) in 0.75 mL of DI water. An amount of 0.05 mmol tin(IV) chloride pentahydrate (${\mathrm{SnCl}}_{4}\cdot \;5H_{2}O$) was dissolved in 6 mL glacial acetic acid under stirring for 10 min. After that, the two prepared solutions were mixed well, and 0.75 mL of ethanol was added into the mixture. Then, the cleaned substrates were loaded into the Teflon-lined autoclave reactor and heated in a muffle furnace at 200 °C for 12 h. The samples with SnO_2_ nanorod arrays were finally cleaned by DI water and ethanol in an ultrasonic bath.

### 2.2. Preparation of SnO_2_ Nanorod Arrays Using Different Growth Parameters

#### 2.2.1. Growth Pressure

The growth pressure was varied by adding the same amount of SnO_2_ precursor solution (7.5 mL) into different volume sizes (25 mL, 50 mL, and 100 mL) of Teflon-lined autoclaves. The filling ratios of the reactors were 30%, 15%, and 7.5%, respectively. The built-up pressure in the reaction system increased with the filling ratios. On the other hand, in order to maintain a constant pressure among different volume sizes of Teflon-lined autoclaves, the same filling ratio of 7.5% was obtained by adjusting the total amount of the precursor solution in each Teflon liner. The compositions of the precursor solution are described in Section 2.1 above.

#### 2.2.2. Substrate Orientation

Three different geometric alignments of the FTO-coated glass substrates were investigated. The substrates with the side of FTO facing down were placed at an angle of 0° (horizontally facing down), 45°, or 90° (vertically orientated) with respect to the bottom surface of the Teflon liner. The polytetrafluoroethylene (PTFE) sample holders were used for holding FTO-coated substrates in place. The filling ratio of the reactor for each condition was maintained at 7.5% using the 100 mL the Teflon-lined autoclaves.

#### 2.2.3. DI Water-to-Ethanol Ratios

The DI water-to-ethanol ratio was adjusted to 1:9, 3:7, 1:1, 7:3, and 9:1, where the 1:1 ratio was referred to a mixture of 0.75 mL of DI water and 0.75 mL of ethanol (1.5 mL in total). The other ratios of DI water to ethanol were varied according to obtain a 1.5 mL mixture. The same amount of glacial acetic acid (6 mL), ${\mathrm{SnCl}}_{4}\cdot \;5H_{2}O$ (0.05 mmol), and NaBr (0.15 mmol) were used for all conditions of different DI water-to-ethanol ratios.

#### 2.2.4. Seed Layers

Three different types of SnO_2_ seed layers composed of thin films, nanoparticles, or quantum dots were prepared on FTO-coated glasses for subsequent solvothermal growth of SnO_2_ nanorod arrays. The SnO_2_ thin films were prepared by the magnetron sputtering. The SnO_2_ ceramic disc (99.99% purity, Kurt J. Lesker Company, Pittsburgh, PA, USA) with a diameter of 50.8 mm was used as a target. A working pressure of 5 × 10^−3^ Torr in a pure argon atmosphere was maintained during the radio frequency sputtering to achieve a SnO_2_ layer in 10 nm. For SnO_2_ quantum dots, the precursor solution was prepared by dissolving 3.99 mol SnCl_2_·2H_2_O in DI water. The solution was stirred for 3–4 h under oxygen flow (1L/min), followed by filtering with a syringe filter with a pore size of 0.45 μm. The prepared solution was spin-coated on top of the FTO-coated glass substrates at 3000 rpm for 30 s. After deposition, the substrates were placed onto a hotplate and annealed at 200 °C for 1 h. For SnO_2_ nanoparticles, the solution was prepared using the SnO_2_ colloidal dispersion solution (Tin(IV) oxide, 15% in H_2_O colloidal dispersion, Alfa Aesar, Heysham, United Kingdom) diluted with DI water in a 1:5 volume ratio. The dispersion solution was spin-coated on top of the FTO-coated glass substrate at 3000 rpm for 30 s, followed by thermal annealing on a hotplate at 150 °C for 30 min. The SnO_2_ precursor solution used for solvothermal growth is described in Section 2.1, except that 6.75 mL instead of 6 mL glacial acetic acid was added into the mixture. The filling ratio of the reactor was 8.3%.

#### 2.2.5. Glacial Acetic Acid

The precursor solution of SnO_2_ was prepared by the procedure as described in Section 2.1 (1:1 DI water-to-ethanol ratio). The glacial acetic acid was used in an amount of 6 mL, 6.5 mL, and 9.75 mL. The filling ratio of the reactor was 7.5%, 8%, and 11.3%, respectively.

#### 2.2.6. Growth Time

The substrates with a 15 nm SnO_2_ thin film as a seed layer were placed in the precursor solution as described in Section 2.1, except that 6.75 mL instead of 6 mL glacial acetic acid was added into the mixture for solvothermal growth of SnO_2_ nanorod arrays. The growth temperature was set as 200 °C for a duration of 6, 12, and 24 h. Meanwhile, another growth condition was designed as a duration of 12 h followed by a replacement of the original precursor with the freshly prepared solution for an additional 12 h growth.

### 2.3. The Perovskite Solution

The perovskite precursor solution was prepared by dissolving 1.2 M PbI_2_ (99%, Sigma Aldrich), 1.1 M FAI (Greatcell Solar Materials, Queanbeyan East, NSW, Australia), 0.2 M PbBr_2_ (≥98%, Sigma Aldrich, St. Louis, MO, USA), 0.2 M MABr (Greatcell Solar Materials, Queanbeyan East, NSW, Australia), and 0.4 M MACl (≥99%, Merck, St. Louis, MO, USA) in 1 mL of a mixture of N,N-Dimethylformamide (DMF, 99.8%, anhydrous, Sigma Aldrich, St. Louis, MO, USA) and dimethyl sulfoxide (DMSO, ≥99.9%, anhydrous, Sigma Aldrich, St. Louis, MO, USA) (4:1 by volume ratio). The solution was stirred on a vortex mixer for 5 h and filtered using the syringe filters with a pore size of 0.45 μm. Then, 28 μL of 1.5 M CsI solution in DMSO and 28 μL of 1.5 M RbI solution in a mixture of DMF:DMSO (4:1 volume ratio) was added to 940 μL of the filtered precursor solution. The perovskite precursor solution was spin-coated on the top of the optimized SnO_2_ nanorod arrays, which were treated by oxygen plasma for 15 s at a power of 70 W using Plasma Prep III Solid State system from SPI Supplies, using a two-step spinning recipe consisting of 1000 rpm for 10 s and 5000 rpm for 30 s. During the last 10 s of the second spinning step, the antisolvent, chlorobenzene (CB, 99.8%, anhydrous, Sigma Aldrich, St. Louis, MO, USA), was dropped onto the sample. Further, the films were placed onto a hotplate and annealed at 105 °C for 75 min. The hole-transport layer (HTL) was prepared by spin coating (3000 rpm for 30 s) the solution composed of 80 mg of 2,2′,7,7′-Tetrakis(N,N-di-p-methoxyphenylamino)-9,9′-spirobifluorene (Spiro-MeOTAD, >99.5%, Lumtec, New Taipei City, Taiwan) dissolved in 954 μL of CB. The solution was doped with 29 μL of 4-tert butylpyridine (TBP, 98%, Sigma Aldrich, St. Louis, MO, USA) and 17 μL of a lithium salt solution. The lithium salt solution was prepared by dissolving 520 mg of Bis(trifluoromethane)sulfonimide lithium salt (Li-TFSI, 99.95%, trace metal basis) in 1 mL of acetonitrile (99.8%, anhydrous, Sigma Aldrich, St. Louis, MO, USA). All the processes as mentioned above were performed in a nitrogen-filled glovebox. Finally, a thickness of 70 nm gold electrode was thermally evaporated on the top of the HTL under the vacuum of 10^−6^ Torr.

## 3. Results and Discussions

It is important to understand the effect of each solvothermal growth parameter on the morphology of SnO_2_ nanostructures. It is noteworthy that some growth parameters have a correlation with each other. For example, varying the volume amount of acetic acid leads to a change in the total volume of precursor solution, resulting in a change of the solvothermal growth pressure. Despite keeping the same molar ratio among the chemicals, a change of the total volume of precursor solution in Teflon-lined autoclaves will affect the growth pressure as well as the absolute amount of reactants available for growing SnO_2_ nanostructures. A systematic study of different growth conditions should be performed experimentally so as to pinpoint their impact on the morphology of the obtained SnO_2_ nanostructures, and thus the reproducibility of desired SnO_2_ nanostructures can be ensured for certain applications.

**Figure 1:**
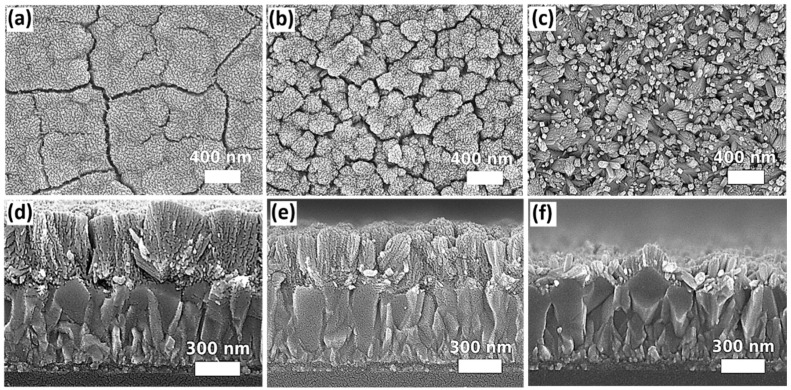
SEM top-view and cross-sectional images of SnO_2_ nanostructures grown in different volume sizes of autoclaves: (**a**,**d**) 25 mL, (**b**,**e**) 50 mL, and (**c**,**f**) 100 mL.

The pressure effect was firstly investigated by using different sizes of Teflon-lined autoclave reactors (25 mL, 50 mL, and 100 mL), filling them with an identical volume and composition of precursor solutions for solvothermal growth at 200 °C for 12 h. The built pressure inside the reactor during solvothermal growth is inversely proportional to the volume size of the Teflon liner. By using a pressure gauge connected to the autoclave reactor during the solvothermal growth process, the pressure was stabilized at 130.0 psi, 87.0 psi, and 72.5 psi for a filling ratio of 30%, 15%, and 7.5%, respectively. Figure 1 shows the top-view and cross-sectional images obtained from the scanning electron microscope (SEM). It is clearly observed that the change of pressure significantly affects the morphology of SnO_2_. The nanorod arrays, in which some of the nanorods are in a small bundle, the structure can be distinguished from the sample prepared in the largest size of the reactor (100 mL). When the pressure is increased by using the smaller reactors, the size of the nanorod bundle increases substantially. Furthermore, the thickness of the nanostructures grown on the FTO-coated glass increases with the growth pressure. Based on the cross-sectional SEM images (Figure 1d–f), an average of thickness was determined as ~425 ± 21 nm, ~319 ± 19 nm, and ~131 ± 12 nm for the samples prepared in 25 mL, 50 mL, and 100 mL reactors, respectively. The difference in nanostructures among the samples prepared in different pressure is due to the changes in Gibbs free energy [65]. A rise in pressure during solvothermal growth leads to an increase of crystallite sizes [65] and induces the coalescence of nanorods to form a bigger bundle [66]. The statistics of the bundle diameters and the length of the SnO_2_ nanorod arrays prepared in different sizes of autoclave reactors are summarized in Table S1 (Supplementary Materials).

**Figure 2:**
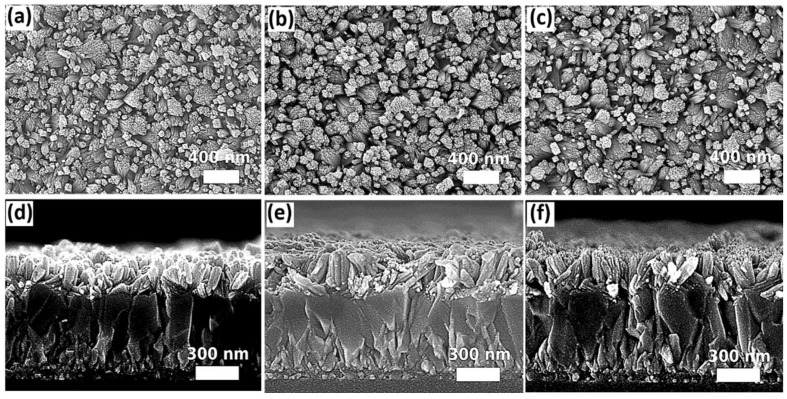
SEM top-view and cross-sectional images of the of SnO_2_ nanostructures grown under the same pressure via adjusting the total volume of the precursor solution to achieve the same filling ratio for three different sizes of autoclave reactors: (**a**,**d**) 25 mL, (**b**,**e**) 50 mL, and (**c**,**f**) 100 mL.

In order to obtain the desired morphology of SnO_2_ nanostructures for device fabrication, the volume of precursor for solvothermal growth should be adjusted accordingly with the size of the autoclave reactors so that an optimized growth pressure can be achieved. However, as mentioned earlier, the precursor solutions in different volumes contain a different total amount of solutes available for reaction, which may probably vary the morphology of the solvothermally grown samples. In order to verify this effect, the volume of the precursor solutions was adjusted accordingly with three different sizes of autoclave reactors to achieve the identical filling ratio (i.e., 7.5%), and hence the same pressure, during solvothermal growth for 12 h. The filling ratio of 7.5% was selected to be investigated further as the SnO_2_ nanorod arrays grown under this condition are less compact, which allows for the penetration of the subsequent deposited perovskite material into the gaps among SnO_2_ nanorods, and thus this structure can facilitate the carrier transport between the SnO_2_ ETL and perovskite absorber. The SEM images of the samples grown in 25 mL, 50 mL, and 100 mL autoclave reactors under the same pressure via adjusting the volume of precursor solution are shown in Figure 2. For a duration of the 12-hour reaction, no significant difference was observed in terms of the surface morphology and lengths of SnO_2_ nanorods, indicating that the same SnO_2_ nanostructure can be reproduced under the same growth pressure regardless of different volumes of precursor solutions (i.e., different total amount of solutes) in this case.

**Figure 3:**
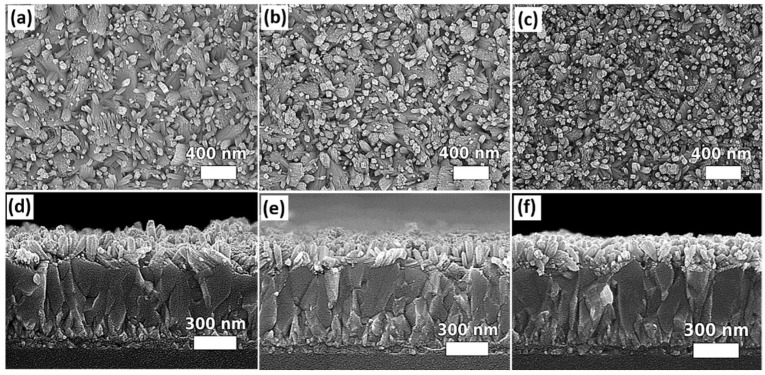
SEM top and cross-sectional images of samples mounted in various orientations for solvolthermal growth: (**a**,**d**) 45°, (**b**,**e**) 90°, and (**c**,**f**) 0°.

The impact of substrate orientation in the autoclave reactor during solvothermal growth on the morphology of the SnO_2_ nanostructures is another concern. The experiment was designed for three different substrate alignments in 100 mL autoclave reactors. The substrates with the side of FTO facing down were placed at an angle of 0°, 45°, and 90° with respect to the bottom surface of the Teflon liner. The SEM images of the samples aligned in different orientations for solvothermal growth are shown in Figure 3. A subtle difference in the morphology of SnO_2_ nanostructures can be observed from the top-view SEM images. For the sample mounted horizontally with the FTO side facing down towards the bottom surface of the Teflon liner (0°), as observed from the top-view SEM image (Figure 3c), relatively more tips of the nanorods point in a perpendicular direction with respect to the surface of the substrate. The density of perpendicularly grown nanorods is reduced, as observed from Figure 3a,b, when the samples were aligned at an angle of 45° or 90°. Instead, the nanorods tend to grow in different directions and form relatively larger bundles compared to the case as shown in Figure 3c (mounted at 0°). The length of SnO_2_ nanorod arrays is shortest for the sample mounted at an angle of 90° (116 nm ± 21 nm) compared to the samples mounted at an angle of 0° (132 nm ± 13 nm) or 45° (135 nm ± 30 nm). Table S2 summarizes the dimensions of SnO_2_ nanostructures grown on the substrates mounted at different orientations. Considering for the slight difference in morphology, the atomic force microscopy (AFM) was performed on the samples mounted at 45°, 90°, and 0° in order to further distinguish their surface characteristics. The obtained results are shown in Figure S1 in the Supplementary Materials. The results show that the root mean square (RMS) roughness is 0.09 μm, 0.04 μm, and 0.07 μm for the samples mounted at 45°, 90°, and 0°, respectively. In this work, placing the substrate horizontally facing down is more practical than other orientations (45° or 90°) as the latter orientations have more restrictions in the dimensions of substrates based on the design of the autoclave reactors. The substrates that are larger in size cannot be fully immersed into the precursor solution, resulting in non-uniform growth of the SnO_2_ nanostructures. Therefore, for further studies of other growth parameters, the substrates were mounted horizontally (0°) for solvothermal growth.

**Figure 4:**
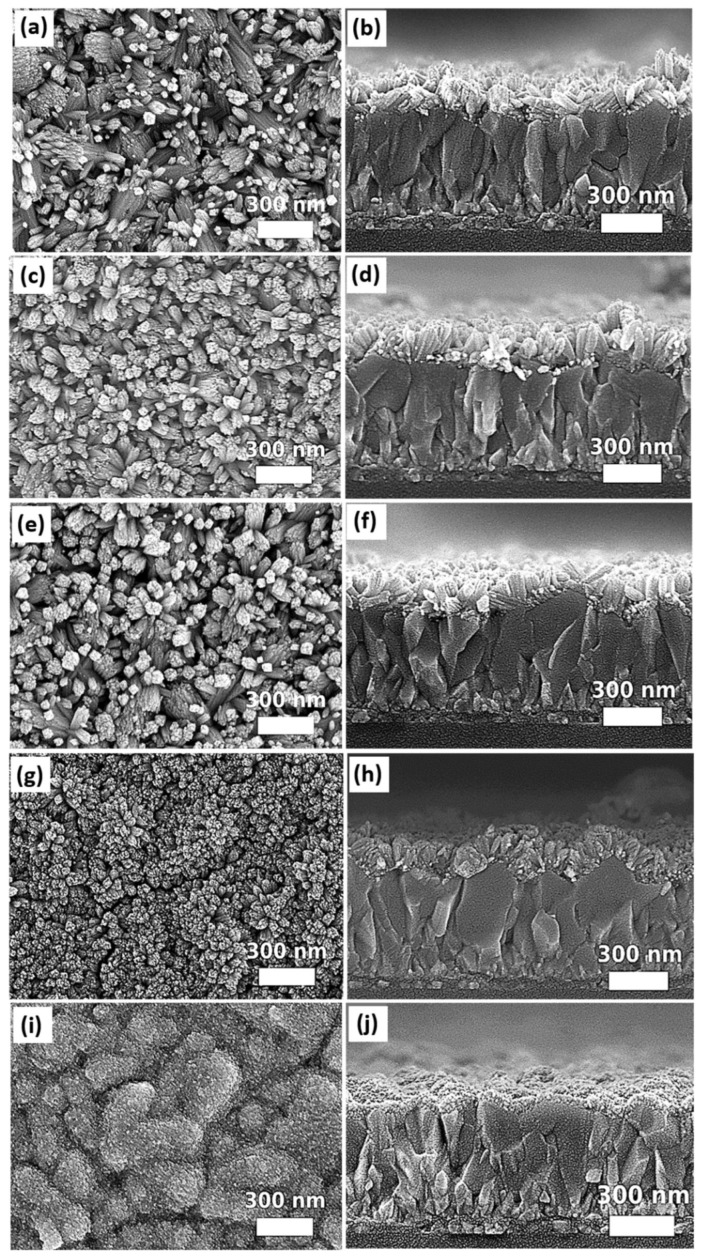
SEM top and cross-sectional images of samples grown in a ternary solvent system with different DI water-to-ethanol ratios: (**a**,**b**) 1:9, (**c**,**d**) 3:7, (**e**,**f**) 1:1, (**g**,**h**) 7:3, and (**i**,**j**) 9:1.

The acetic acid, ethanol, and DI water form a ternary solvent system for the solvothermal growth. The work of Chen et al. [64] demonstrated that the appropriate mixing ratios of these three components are necessary to obtain SnO_2_ nanorod arrays on Ti foil. In this work, the recipe of solvothermal growth of SnO_2_ nanorod arrays reported by Chen et al. [64] was modified accordingly with the size of autoclave reactors and designed experimental conditions. It is noteworthy that the optimization of a ternary solvent system can be complicated as the observed effect from solvothermally grown samples is a result of the interplay of three solvents. Therefore, in this study, the impacts of DI water, ethanol, and the acetic acid on the morphologies of SnO_2_ nanostructures were investigated separately via systematically varying the volume amount of each solvent. The demonstration of the solvent effects on SnO_2_ nanostructures was based on FTO-coated glass substrates, which are the common substrates used for PSCs. Figure 4 shows the top-view and cross-sectional images of the samples prepared by using different DI water-to-ethanol ratios for solvothermal growth. A 1.5 mL solvent mixture prepared by mixing the DI water and ethanol in different volume ratios (1:9, 3:7, 1:1, 7:3, or 9:1) was used for solvothermal growth, while other constituents of the precursor solution were kept constant in a 100 mL Teflon liner. The DI water involves the hydrolysis process of ${\mathrm{SnCl}}_{4}\cdot \;5H_{2}O$ while ethanol facilitates the formation of a well-defined SnO_2_ nanorod array [64]. It is known that increasing water content can accelerate the hydrolysis process of ${\mathrm{SnCl}}_{4}\cdot \;5H_{2}O$. It is consistent to the observation that, when DI water-to-ethanol ratio is 1:9, the SnO_2_ in form of nanorods can be distinguished. Chen et al. [64] reported that low water content causes slow hydrolysis, which leads to formation of small cube-like nanoparticles on the substrate after 24 h solvothermal growth. Compared to our results, it is believed that the amount of the smallest portion of water used in this work is still within the tolerance for growing SnO_2_ nanorods. On the other hand, the varying amount of ethanol demonstrates a significant impact on the morphology of SnO_2_ nanostructures. For the condition of using a very low portion of ethanol (DI water-to-ethanol ratio: 9:1), a thin layer composed of nanoparticles grown on the FTO can be observed (Figure 4i,j). This is consistent with the results reported by Chen et al. [64], for which a lower ethanol content leads to the growth of SnO_2_ nanoparticles instead of nanorods. Furthermore, a trend of transformation from nanoparticles to distinguishable nanorods can be observed from our results when comparing the SEM images of the samples prepared in the solvent with an increasing proportion of ethanol.

It is interesting to observe that the nanorods tend to form larger bundles when the samples are prepared at a condition of the highest ethanol proportion (Figure 4a), compared to other samples grown at the conditions with a lower proportion of ethanol (Figure 4c,e). For the condition of DI water-to-ethanol ratio at 7:3, the nanorod structures start to be barely distinguishable from the top-view SEM image (Figure 4g), while the dense nanostructures composed of clusters and nanorods can be observed from the corresponding cross-sectional SEM image (Figure 4h), which is the result of the interplay between the relatively high portion of DI water and moderately low portion of ethanol. It is noteworthy that the differences in the molecular weight and boiling point of the DI water and ethanol can also affect the vapor pressure. Therefore, the pressure built in the autoclave reactor for the conditions of using 1:9, 1:1, and 9:1 DI water: ethanol ratio was measured by a pressure gauge. It is found that a similar pressure of 72.5 psi was detected for all testing conditions. This result indicates that the pressure effect associated with variations of the DI water-to-ethanol ratio on the change of SnO_2_ nanostructures, as shown in Figure 4, is negligible under current experimental conditions. The dimensions of SnO_2_ nanostructures grown in a ternary solvent system with different DI water-to-ethanol ratios are summarized in Table S3 in Supplementary Materials. The ratio of DI water to ethanol was selected as 1:1 for further study of other solvothermal growth parameters.

**Figure 5:**
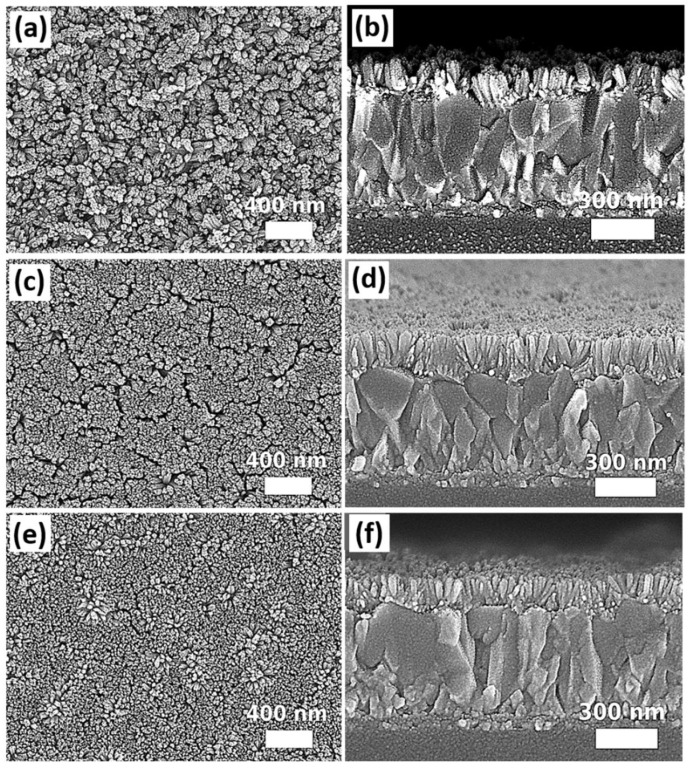
The top-view and cross-sectional SEM images of SnO_2_ nanorod arrays grown on different types of seed layers: (**a**,**b**) SnO_2_ thin film prepared by the magnetron sputtering, (**c**,**d**) SnO_2_ nanoparticles prepared by spin coating of suspension solution, and (**e**,**f**) SnO_2_ quantum dots prepared via a sol-gel process.

Usually, a compact ETL is used underneath the metal oxide nanostructures. The usage of the compact ETL can avoid forming the shunt paths due to the direct contact of the perovskite layer with the conductive FTO through the gaps of the nanostructures. In this work, three different types of compact layers, which can also act as a seed layer for subsequent solvothermal growth of SnO_2_ nanostructures, were prepared by magnetron sputtering, spin coating of the SnO_2_ nanoparticle (10–15 nm) dispersion solution, and deposition of SnO_2_ quantum dots (~5 nm) via a sol-gel process [12]. The top-view and the cross-sectional images of SnO_2_ nanostructures grown on different types of seed layers are shown in Figure 5.

Comparing to the samples of SnO_2_ nanostructures grown on FTO without a seed layer, it can be noticed that the nanorods grown on the seed layer tend to orientate in a single direction perpendicular to the substrates. Furthermore, the density of the nanorod arrays is obviously increased when they were grown on the seed layer regardless of their types. Ideally, SnO_2_ nanorod arrays used as ETL for perovskite solar cells should contain reasonable free space among the nanostructures to accommodate the perovskite grains. The structure of perovskite grains embedded in SnO_2_ nanorod arrays increases the interfacial areas between the two materials so that the photogenerated electrons can be transported efficiently between the perovskite and ETL before carrier recombination. In this work, only SnO_2_ nanorod arrays grown on the SnO_2_ thin film prepared by the magnetron sputtering exhibit more free spaces, as observed from Figure 5a,b. Besides, compared to the samples of using the other two types of seed layers, the diameters of the nanorods grown on the SnO_2_ thin film deposited by the magnetron sputtering seem to be the largest, as more SnO_2_ nanorods join to form a larger bundle. Based on the SEM images of Figure 5e,f, SnO_2_ quantum dots induce the growth of SnO_2_ nanorods in the highest density and finest diameter, resulting in the highest compacity of SnO_2_ nanostructures. The SnO_2_ nanorods grown on SnO_2_ nanoparticles are relatively larger in diameter and form relatively less compact nanostructures (Figure 5c,d) compared to the nanorods grown on SnO_2_ quantum dots. It is believed that the size of the SnO_2_ particles will affect the density of the nucleation sites, leading to the difference in morphology of SnO_2_ nanorod arrays grown subsequently. Nevertheless, varying SnO_2_ quantum dots and nanoparticle concentrations in the seed layer should be performed further to investigate their impact on the morphology of subsequently grown nanomaterials. The dimensions of SnO_2_ nanostructures grown on different types of seed layers are summarized in Table S4 (Supplementary Materials). Considering the criteria of ETL used in PSCs, it is more suitable to prepare the compact layer by the magnetron sputtering, as the morphology of SnO_2_ nanorod arrays grown on the top contains more free spaces for subsequent growth of perovskite grains.

**Figure 6:**
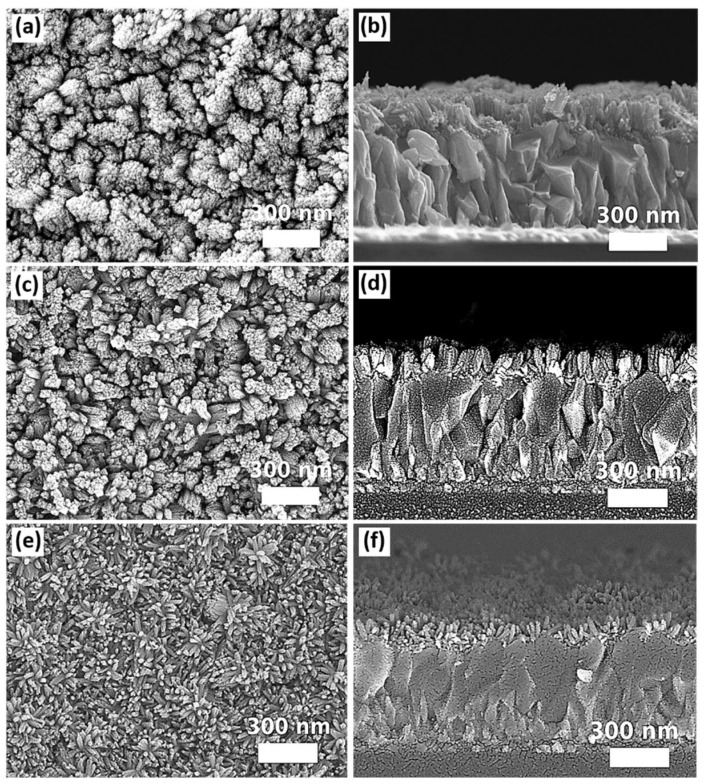
The top-view and cross-sectional SEM images of SnO_2_ nanostructures prepared from the precursor solution containing (**a**,**b**) 6 mL, (**c**,**d**) 6.5 mL, and (**e**,**f**) 9.75 mL of acetic acid.

The impact of different amount of acetic acid (6 mL, 6.5 mL, and 9.75 mL) on the morphology of SnO_2_ nanostructures was investigated based on the samples with a seed layer prepared by the magnetron sputtering. The differences in obtained morphology of SnO_2_ nanorod arrays are shown in Figure 6. It is found that when the amount of acetic acid is reduced from 9.75 mL to 6 mL, SnO_2_ nanorods tend to form increasing bundle sizes and lengths. Chen et al. [64] suggested that acetic acid can reduce the rate of hydrolysis due to the coordination of CH_3_COO^−^ ions with Sn^4+^ ions. Meanwhile, the acetic acid and its side product ethyl acetate, generated during solvothermal growth, are the organic ligands, which can promote the growth of well-defined tetragonal SnO_2_ nanorods and prevent the fusion of SnO_2_ nanorods into bundles. Our experimental results are consistent with the proposed mechanism of Chen et al. It is observed from the SEM images (Figure 6b,d) that the lengths of SnO_2_ nanorods prepared at the condition with 6 or 6.5 mL acetic acid are significantly longer than the samples prepared at the condition using 9.75 mL acetic acid, which can be probably attributed to the higher hydrolysis rate during solvothermal growth because of using a lower amount of acetic acid (6 mL and 6.5 mL). On the other hand, when a lower amount of acetic acid was used (6 mL), SnO_2_ nanorods were merged together to form larger bundles, as observed from Figure 6a,b. When the amount of acetic acid was increased from 6 to 6.5 mL, the sizes of SnO_2_ nanorod bundles were significantly reduced with more free spaces generated among the nanostructures (Figure 6c,d). When the amount of acetic acid (9.75 mL) was further increased, SnO_2_ nanorods became more distinguishable and well separated from each other, while their lengths were the shortest (80 nm ± 17 nm) among all conditions using different amounts of acetic acid. The lengths of SnO_2_ nanostructures grown in different acetic acid concentrations are summarized in Table S5 (Supplementary Materials). Our results clearly demonstrate the role of acetic acid in controlling the nanomorphology of solvothermally grown SnO_2_ nanostructures. It is noteworthy that using different amounts of acetic acid also varies the volume of precursor solution, which contributes to the change of growth pressure. However, in this case, it is not possible to decouple pressure changes by varying the amount of acetic acid. Nevertheless, the impact of pressure changes attributed to varying the volume of acetic acid from 6 mL to 9.75 mL on the morphology of SnO_2_ nanostructures is relatively small compared to the conditions for preparing the samples, as shown in Figure 1.

**Figure 7:**
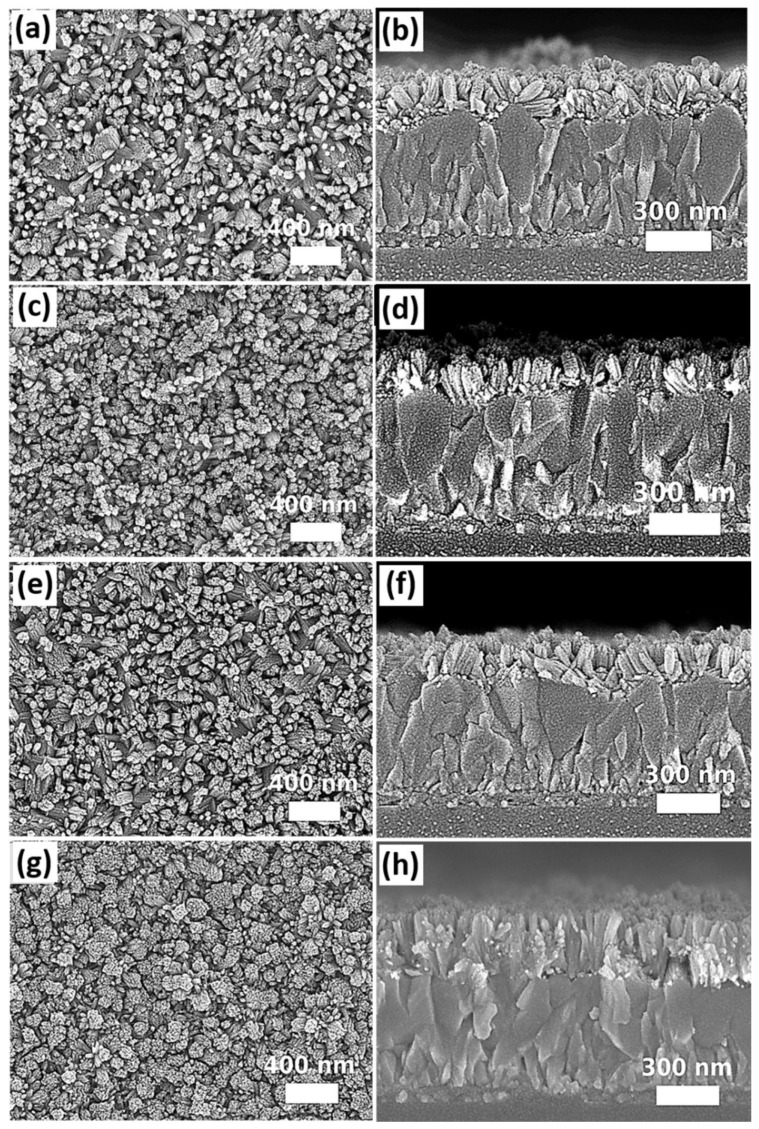
The top-view and cross-sectional SEM images of SnO_2_ grown by solvothermal method for (**a**,**b**) 6 h, (**c**,**d**) 12 h, (**e**,**f**) 24 h, and (**g**,**h**) 12 + 12 h (two-step process).

Further investigation of growth time was performed based on the experimental condition for preparing samples, as shown in Figure 6 b,c. The growth temperature was set as 200 °C for different durations (6, 12, and 24 h). Besides, another growth condition was designed as a duration of 12 h followed by a replacement of the original precursor with the freshly prepared solution for an additional 12 h growth. The top-view and cross-sectional SEM images are shown in Figure 7. By comparing the SEM images among Figure 7a–f, different growth durations (6, 12, and 24 h) do not cause significant changes in terms of SnO_2_ nanostructures as well as the length of the nanorods, as observed from the top-view and cross-sectional SEM images of the samples. However, if a two-step process is implemented by performing consecutive solvothermal growth two times with the use of freshly prepared precursor solutions, the obtained SnO_2_ nanostructures become more compact with larger size of nanorod bundles. Meanwhile, the length of nanorods prepared by the two-step process exhibit an increase of ~1.7 times (i.e., 219 nm ± 49 nm) compared to the length of SnO_2_ nanorods prepared by the one-step solvothermal growth process. The summary of the dimensions of SnO_2_ nanostructures grown with different growth duration is shown in Table S6 (Supplementary Materials). It is believed that the non-sensitivity of SnO_2_ nanostructures towards changing of different growth durations in the one-step solvothermal growth process is due to the other limiting factors such as the total amount of different solutes in the precursor solution. Based on our experiments, it is challenging to vary the length of the nanorods as an independent parameter while keeping the desired density and dimension of SnO_2_ nanorods/bundles unchanged. Further intensive research efforts should be placed on different combinations of experimental conditions. Nevertheless, our obtained results so far demonstrate the general trend of morphological changes in SnO_2_ nanostructures caused by different solvothermal growth parameters.

**Figure 8:**
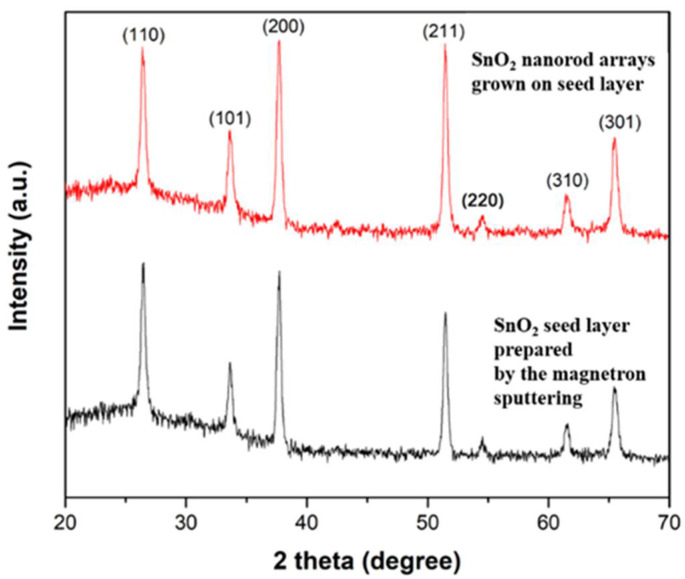
The XRD patterns of the SnO_2_ seed layer prepared by the magnetron sputtering and solvothermally grown SnO_2_ nanorod arrays.

**Figure 9:**
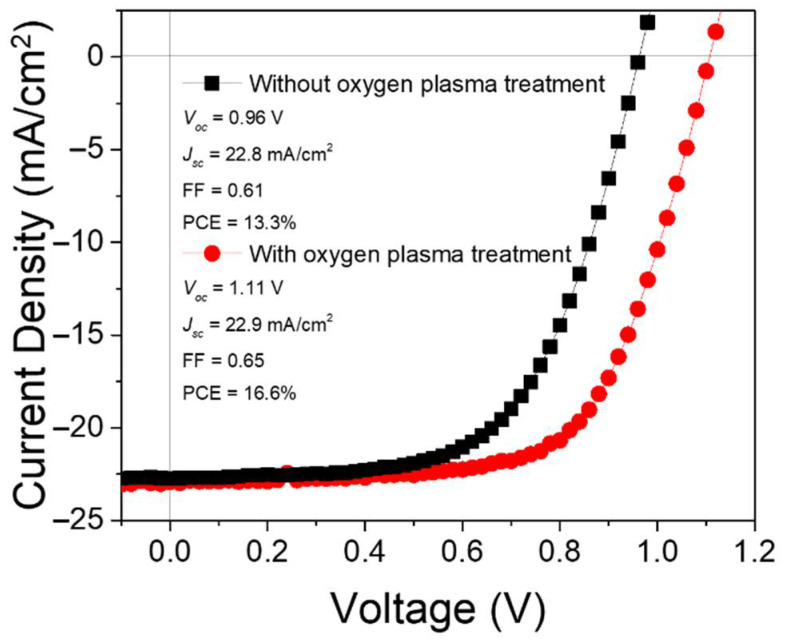
The *J-V* characteristics of the PSCs based on SnO_2_ nanorod arrays with and without oxygen plasma treatment.

The SnO_2_ nanorod arrays can be used as the ETL in PSCs. The samples with the similar morphology of SnO_2_ nanorod arrays, as shown in Figure 7c,d, were used for device fabrication. The purity of SnO_2_ nanostructures used for device fabrication was confirmed by the X-ray diffractometer. The obtained XRD patterns of the SnO_2_ seed layer prepared by the magnetron sputtering and SnO_2_ nanorod arrays grown on the seed layer are shown in Figure 8. The detected XRD peaks were consistent to the reported work [67] showing the tetragonal SnO_2_ material. The *I-V* characteristics of the fabricated PSCs were studied. The obtained results are plotted in Figure 9. It is found that the PSC with SnO_2_ ETL treated by oxygen plasma exhibits significantly improved photovoltaic parameters compared to the device without this surface treatment on the ETL. The removal of the organic residuals from the surface of SnO_2_ nanostructures is believed to be the reason for the enhancement of device performance. This finding demonstrates that a proper surface treatment on solvothermally grown SnO_2_ nanostructures is important for the fabrication of PSCs. It is noteworthy that there is a strong interplay between the SnO_2_ nanostructures and the perovskite layer deposited above. Therefore, each type of SnO_2_ nanostructure requires individual optimization of perovskite layer as well as their material interface (ETL/Perovskite) in order to achieve the best processing condition for a certain combination of SnO_2_ nanostructured ETLs and perovskite layers, resulting in optimized PSCs. However, these topics are out of the scope of this work.

## 4. Conclusions

The impacts of different growth parameters including growth pressure, substrate orientation, DI water-to-ethanol ratios, types of seed layer, amount of acetic acid, and growth time on the morphology of solvothermally grown SnO_2_ nanostructures were systematically investigated. It was found that the volume of the precursor solution should be adjusted according to the size of the autoclave reactors to obtain the optimized growth pressure. The orientation of substrates can affect the growth direction of SnO_2_ nanorods. The ternary solvent system composed of DI water, ethanol, and acetic acid should be optimized in terms of their volume ratio, which can affect the density of the SnO_2_ nanorods and their bundle size. The presence of the seed layer on FTO significantly affects the morphology of SnO_2_ nanostructures. The SnO_2_ nanoparticle-based seed layers tend to induce more compact growth of SnO_2_ nanorod arrays compared to the sample using the seed layer prepared by the magnetron sputtering. The length of SnO_2_ nanorods can be increased by using a two-step solvothermal growth method while keeping other morphological properties of SnO_2_ nanorod arrays constant, along with increasing thickness, is still challenging. For employing solvothermally grown SnO_2_ nanostructures as an ETL in PSCs, a proper surface treatment such as oxygen plasma should be performed on the SnO_2_ ETL in order to achieve improved photovoltaic performance of the devices.

## Data Availability

The authors confirm that the data supporting the findings of this study are available within the article and its Supplementary Materials.

## Supplementary Materials

The following supporting information can be downloaded at: https://www.mdpi.com/article/10.3390/nano12101686/s1, Table S1: The dimensions of SnO_2_ nanostructures grown in different pressure by using different sizes of autoclave reactors; Table S2: The dimensions of SnO_2_ nanostructures grown on substrates mounted at different orientations; Table S3: The dimensions of SnO_2_ nanostructures grown in a ternary solvent system with different DI water to ethanol ratios; Table S4: The dimensions of SnO_2_ nanostructures grown on different types of seed layers; Table S5: The dimensions of SnO_2_ nanostructures grown in different acetic acid concentrations; Table S6: The dimensions of SnO_2_ nanostructures grown with different growth durations; Figure S1: The topography obtained by the atomic force microscopy for the samples mounted in various orientations during solvothermal growth durations.
