# Supplementary material for: A Morphological Study of Solvothermally Grown SnO2 Nanostructures for Application in Perovskite Solar Cells
Source: Nanomaterials (Basel). 2022 May 15;12(10):1686. doi: 10.3390/nano12101686 (PMC9143344; doi:10.3390/nano12101686)
Supplement: Supplementary file 1 [file nanomaterials-12-01686-s001.zip › nanomaterials-1655076-supplementary.pdf]

nanomaterials

Supplementary Information

A Morphological Study of Solvothermally Grown SnO_2 Nanostructures for Application in Perovskite Solar Cells

Zhuldyz Yelzhanova [1,†], Gaukhar Nigmetova [2,†], Damir Aidarkhanov [1], Bayan Daniyar [1], Bakhytzhan Baptayev [3], Mannix P. Balanay [4], Askhat N. Jumabekov [5] and Annie Ng [1,*]

[1] Department of Electrical and Computer Engineering, School of Engineering and Digital Sciences, Nazarbayev University, Kabanbay Batyr Ave. 53, Nur-Sultan 010000, Kazakhstan; zhuldyz.yelzhanova@nu.edu.kz (Z.Y.); aidarkhanov@nu.edu.kz (D.A.); bayan.daniyar@nu.edu.kz (B.D.)
[2] Department of Chemical and Materials Engineering, School of Engineering and Digital Sciences, Nazarbayev University, Kabanbay Batyr Ave. 53, Nur-Sultan 010000, Kazakhstan; gaukhar.nigmetova@nu.edu.kz
[3] National Laboratory Astana, Kabanbay Batyr Ave. 53, Nur-Sultan 010000, Kazakhstan; bbaptayev@nu.edu.kz
[4] Department of Chemistry, School of Sciences and Humanities, Nazarbayev University, Kabanbay Batyr Ave. 53, Nur-Sultan 010000, Kazakhstan; mannix.balanay@nu.edu.kz
[5] Department of Physics, School of Sciences and Humanities, Nazarbayev University, Kabanbay Batyr Ave. 53, Nur-Sultan 010000, Kazakhstan; askhat.jumabekov@nu.edu.kz
* Correspondence: annie.ng@nu.edu.kz
† These authors contributed equally to this work.

Table S1. The dimensions of SnO_2 nanostructures grown in different pressure by using different sizes of autoclave reactors.

Condition	Length (nm)			Diameter of SnO_2 Bundle (nm)		
-	*Min* (nm)	*Max* (nm)	*Mean ± SD* (nm)	*Min* (nm)	*Max* (nm)	*Mean ± SD* (nm)
**25 mL, 130 psi**	383	478	425±21	1092	1484	1220±140
**50 mL,87 psi**	278	349	319±19	244	572	389±82
**100 mL,72.5 psi**	108	156	131±12	40	112	60±21

Table S2. The dimensions of SnO_2 nanostructures grown on substrates mounted at different orientations.

Condition	Length (nm)			Diameter of SnO_2 Bundle (nm)		
-	*Min* (nm)	*Max* (nm)	*Mean ± SD* (nm)	*Min* (nm)	*Max* (nm)	*Mean ± SD* (nm)
**45°**	87	178	135±30	42	156	69±28
**90°**	86	154	116±21	42	185	91±41
**0°**	105	152	132±13	46	91	64±14

Table S3. The dimensions of SnO_2 nanostructures grown in a ternary solvent system with different DI water to ethanol ratios.

Condition	Length (nm)			Diameter of SnO_2 Bundle (nm)		
-	*Min* (nm)	*Max* (nm)	*Mean ± SD* (nm)	*Min* (nm)	*Max* (nm)	*Mean ± SD* (nm)
**1:9**	79	186	139±26	47	215	81±42
**3:7**	103	178	136±18	50	98	73±15
**1:1**	102	154	131±16	45	83	61±11
**7:3**	69	129	100±19	26	70	41±12
**9:1**	30	77	48±14	NA	NA	NA

Table S4. The dimensions of SnO_2 nanostructures grown on different types of seed layers.

Condition	Length (nm)		
-	*Min* (nm)	*Max* (nm)	*Mean ± SD* (nm)
**Magnetron Sputtering**	104	155	128±14
**NP**	102	200	164±26
**QD**	72	136	109±16

Table S5. The dimensions of SnO_2 nanostructures grown in different acetic acid concentrations.

Condition	Length (nm)		
-	*Min* (nm)	*Max* (nm)	*Mean ± SD* (nm)
**6 mL**	91	151	119±16
**6.5 mL**	105	153	128±13
**9.75 mL**	55	119	80±17

Table S6. The dimensions of SnO_2 nanostructures grown with different growth durations.

Condition	Length (nm)			Diameter of SnO_2 Bundle (nm)		
-	*Min* (nm)	*Max* (nm)	*Mean±SD* (nm)	*Min* (nm)	*Max* (nm)	*Mean ± SD* (nm)
**6 hrs**	83	138	119±14	37	109	62±22
**12 hrs**	101	153	129±16	45	91	65±11
**24 hrs**	97	154	130±15	39	146	79±33
**12+12 hrs**	131	312	219±49	56	202	109±40

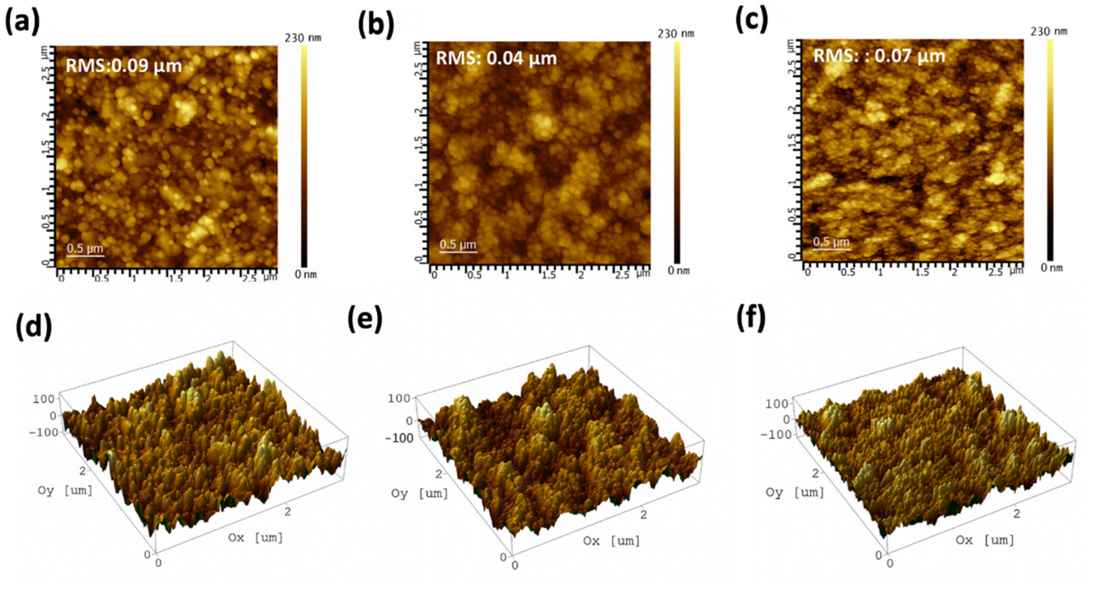

Figure S1. The topography of SnO_2 nanorods obtained by the atomic force microscopy for the samples mounted in various orientations during solvothermal growth: (**a**,**d**) 45°; (**b**,**e**) 90°; (**c**,**f**) 0°.
